# The Position of the Medical Examiner for Life Assurance.—III


**Published:** 1892-07-02

**Authors:** 


### THE POSITION OF THE MEDICAL EXAMINER FOR LIFE ASSURANCE.*—III.

(*Continued.*)

We have already alluded to the necessity of duly considering the value to be attached to varying intensity of phthisical predisposition in estimating expectation of life. There are many points in connection with heart disease which require careful study from the life assurance aspect. Does the presence of a cardiac bruit from valvular imperfection render a life uninsurable? We should say most decidedly not. It not unfrequently happens that men have enjoyed the most perfect health till it has been accidentally discovered that they have a cardiac bruit, and a slight well-compensated mitral regurgitation may be accepted on slightly increased rates, provided the life be in every other respect unexceptionable. The loudness of the bruit is no criterion as to the degree of valvular imperfection. Certain it is that mere valvular disease is of far less import than a diseased condition of the muscular wall, although the first may be so manifest and easily detected, and the latter so extremely indefinite in its symptoms. Aortic stenosis in its slighter degrees is not in itself a very serious defect, while aortic regurgitation, as is well known, is so certain to shorten life materially, that its presence excludes the patient from further consideration for insurance.

Of all points the most open question that at present concerns life insurance in its medical aspects is the advisability of accepting or rejecting albuminuric patients. Ever since Dr. Bright demonstrated the connection between albumen in the urine, and dropsy, and kidney disease, the medical profession have regarded the presence of albumen in the urine as a sufficient indication of renal disease. Recently, however, our ideas as to the meaning of albumen in the urine have undergone considerable modification, and now several offices are prepared to accept, at increased rates, certain classes of albuminuria. We consider that upon life insurance medical officers lies the onus of proving that the presence of albumen in the urine persistently, but in mere traces, is a sufficient indication of an unphysiological condition of kidney, and necessarily tends to shorten life. There is no sufficient reason for starting with the idea that albumen in the urine in the merest trace is pathological, and calling on those who differ from us to prove the contrary.

We cannot enter into this question here, but we should remember that it is not always an easy matter to be quite certain that albumen in the urine comes from the kidney, and it behoves the examiner

* Address to Bristol Insurance Institute, by Watson Williams, M.D.

to ascertain whether the patient is suffering from some temporary defect in the bladder or urethra, whether there is any pus present or discharge which may give rise to unfounded suspicion of albuminuria. And if albumen is present, it is our duty to be most clear as to the condition of the pulse, to examine the eye with an ophthalmoscope to ascertain the condition of the retina, and to report fully on these and many other collateral details, so that the candidate may have his case fairly laid before the medical board, and not suffer needless rejection. These details are frequently omitted, as their value is not always foreseen by the examiner. Now, when an applicant has decided to take out a policy it is well to remember that, as a rule, he has been induced to do so, not of his own free will, but because circumstances render it expedient. Thus it is of great importance that no time be lost before he is examined, otherwise a more persuasive rival agent may step in, or the candidate may change his mind, and an examiner who is always prompt and obliging in this respect is generally appreciated by the office, by the candidate, and last, but not least, his agent. The examiner who is wanting in tact and is deficient in technical skill undoubtedly loses many good lives and admits many bad ones, thus spoiling in both ways the business of the office, defrauding the agent, and tending to lessen public confidence in life assurance. A good office is generally very particular in its selection of medical examiners, for the directors know well enough that in these days of keen competition it cannot afford to lose good business, while bad lives tend to maintain high premiums.

The first actual life insurance company in England was established only so recently as 1714, although the Hand-in-Hand, a fire office, founded in 1696, did some life business too. Yet see to what great importance in social and commercial life your profession has risen. Life insurance is a business contract, and its engagement is not founded on any philanthropic, benevolent, or charitable principles. Now since the very foundation of good business is careful medical selection, is it not truly astounding that so little care is observed in the choice of examiners by many offices? More especially does this apply to the country districts, where the country practitioner cannot have special experience, and where he is placed in a most awkward position with his patient if he decline a life. It would pay any good company over and over again to send out a skilled examiner from the larger centres, at a slightly increased fee, instead of asking the practitioner who is completely at sea in life insurance work, with the result that the agent often loses a good case and the office accepts bad ones. The office that uniformly adopts the course commended will soon find that the business improves in quality, and, consequently, also in quantity. There is nothing more exasperating than for the agent of a good office to feel that a good case has been lost by want of skill or promptitude on the part of the representative examiner, and the more interest medical men take in insurance work and its methods, the more will they recognize and sympathise with the difficulties of life insurance agents, and their reports will be increasingly valuable to the office they represent.

Our remarks on the medical aspect of life assurance are not intended to do more than direct attention to some of the technical questions that arise in making examinations, and to indicate some of the many ways in which the retention of the medical examiner becomes absolutely necessary if good results are to be obtained in connexion with new business. We have also endeavoured to very briefly indicate some of the reasons why the medical examiner has not proved himself indispensable to all the offices for life assurance.

